# What Factors Guide the Selection of Medicinal Plants in a Local Pharmacopoeia? A Case Study in a Rural Community from a Historically Transformed Atlantic Forest Landscape

**DOI:** 10.1155/2018/2519212

**Published:** 2018-01-21

**Authors:** Taline Cristina da Silva, Josilene Marinho da Silva, Marcelo Alves Ramos

**Affiliations:** ^1^Biology Department, University of Alagoas State, BR 316, Km 87,5, Av. Bebedouro, 57500-000 Santana do Ipanema, AL, Brazil; ^2^Laboratory of Ethnobiological Studies (LEET), University of Pernambuco, Campus Mata Norte, Amaro Maltês de Farias St., 55800-000 Nazaré da Mata, PE, Brazil

## Abstract

The criteria that local people use for selecting medicinal plants have been a recurrent topic in pharmacology and ethnobotany. Two of the current hypotheses regarding this phenomenon, ecological apparency and diversification, attempt to explain the inclusion of “apparent” and “non-apparent” and native and exotic taxa, respectively, in local pharmacopoeia. This study addresses the following questions: Do “apparent” and “non-apparent” medicinal plants have the same importance in local pharmacopoeia? Do “non-apparent” plants occupy more local categories of diseases than “apparent” plants? Do native and exotic medicinal plants have the same importance? Do exotic and native plants occupy different local categories of diseases? This study was conducted with householders of a community from Northeastern Brazil. Out of the 66 plant species cited, most were herbs (39 species), followed by trees and shrubs (27). Herbaceous species also occupied more local categories of diseases (51) than tree and shrub species (28). Furthermore, most of the species cited by the informants were exotic (42). Out of the 94 therapeutic applications cited in this research, 65 were treated with exotic species and 29 with native species, distributed among 13 body systems. These results support both the hypotheses of ecological apparency and diversification.

## 1. Introduction

Studies investigating the criteria used by human populations to select plants according to their medical repertoire are recurrent in ethnopharmacology and ethnobotany [1–3]. Therefore, it is not surprising that several hypotheses have been proposed in an attempt to explain the diversity of plant species in pharmacopoeia, among which are the hypothesis of ecological apparency and the hypothesis of diversification.

The hypothesis of ecological apparency was proposed by Feeny [4] and categorizes plant species into “apparent” (e.g., shrubs and trees) and “non-apparent” (e.g., herbs). “Apparent” plant species produce organic compounds of high molecular weight and low toxicity that act as digestive inhibitors in herbivores (quantitative defenses), whereas “non-apparent” plants produce organic compounds of low molecular weight, high toxicity, and high bioactivity (qualitative defenses) [5, 6]. In the human context, this hypothesis was initially proposed by Phillips and Gentry [7]. They proposed that the most abundant species are most often found and, consequently, people have more opportunities to experiment and learn their medicinal uses. In adding plant species into a pharmacopoeia, the hypothesis of ecological apparency assumes that herbaceous plants (non-apparent plants) usually contain chemical compounds that are more bioactive than those found in shrub or tree species (apparent plants). This may explain the greater importance of herbaceous plant species in the pharmacopoeia of various human populations from different Brazilian ecosystems [6, 8, 9]. However, only a few studies, which show contrasting results [10, 11], have addressed the apparency hypothesis in the Brazilian Atlantic Forest, where the validity of this hypothesis is still intensely debated.

Another hypothesis for including medicinal plants in the repertoire of human populations is the hypothesis of diversification, which involves the incorporation of exotic plants into pharmacopoeias [1]. The most accepted explanation for including exotic species into pharmacopoeias is the loss of local knowledge as a result of the transformation of consuetude from rural to urban centers, which promotes changes in the traditional medical systems due to acculturation or erosion of knowledge [9, 12]. However, the hypothesis of diversification predicts that the inclusion of exotic plants diversifies a local therapeutic repertoire, contributing to the treatment of a broader range of therapeutic targets and, in some cases, treats diseases which native species do not [1, 3]. Thus, treating the incorporation of exotic plants into a pharmacopoeia as acculturation and/or loss of knowledge should be viewed with caution because this perspective often ignores the fact that knowledge systems are dynamic and may contain strong adaptive components [13].

The consequences of these two hypotheses can be associated with the transformation of landscapes, since a higher incidence of “non-apparent” (herbs) and exotic plants in local pharmacopoeia can be explained by the use of anthropogenic areas as the main source of plant resources [14]. Some authors have reported that a large proportion of medicinal plants in traditional pharmacopoeia are derived from secondary forests and anthropogenic areas, and exotic herbs tend to dominate the most cited species [15, 16]. Thus, in the context of a transformed landscape, as historically has been the case for the Brazilian Atlantic Forest, this study aims to investigate, using the apparency and diversification hypotheses, the role of “apparent” and “non-apparent” plants and native and exotic plants in the local pharmacopoeia of the rural community of Engenho Cuieiras in the city of Aliança, Pernambuco, Northeastern Brazil. The following questions are addressed: Do “apparent” and “non-apparent” medicinal plants have the same importance? Do “non-apparent” plants make up more local categories of diseases than “apparent” plants? Do native and exotic medicinal plants have the same importance in a local pharmacopoeia? Do exotic and native plants occupy different local categories of diseases?

## 2. Material and Methods

### 2.1. Study Area

The Engenho Cuieiras community was selected as the focus of this research (07°38′ S, 35°14′ W; 150 m; Souza, 2009). The community consists of private property in city of Aliança, Pernambuco, which is located 80 km from Recife, the state capital. It encompasses an area of 266.46 km with a population estimated to be about 37,415 inhabitants [17]. The climate is tropical-rainy, with dry summers and a rainy season that starts in January/February and ends in September/October; the average rainfall is 900 mm per year.

Engenho Cuieiras is surrounded by semideciduous forest fragments belonging to the community, sugarcane plantations, a large house with crops of various food plant species, an area reserved for livestock, and a Municipal School that operates only in the afternoons for literacy. There is also a Child Labor Eradication Program (PETI) operating in the area. The community is comprised of 106 people distributed among 21 households, 17 families in large houses and four families in farm houses. The patriarchs of the families work in the sugarcane plantations, while the women generally assume domestic activities and assist their husbands in activities related to family farming.

### 2.2. Data Collection

Initial visits were made for community recognition and to present the intentions and goals of the research. Prior to these visits, we contacted the local health agent, who provided us with information about the community profile. When residents agreed to participate in the study, they were asked to sign a consent form (TCLE) (Resolution 510/2016, National Health Council), following the ethical regulations of research involving human subjects. In the case of illiterate informants, the term was read in its entirety and if they accepted to participate, they were asked to sign with their fingerprint. This study is part of a larger research project called “Forest Resources in the Area of Pernambuco Forest: Potential Use and Conservation Priorities” submitted to and approved by the Research Ethics Committee of the University of Pernambuco (CAAE 19938013.1.0000.5207).

Interviews were conducted with the heads of family of each household (man and woman); however, there were cases where one of the two refused to participate in the study or was not present during the visits. Thus, 38 interviews were conducted with residents of the 21 residences in the community, 18 men and 20 women between the ages of 19 and 70 years.

The initial part of the data collection was performed using semistructured forms [18]. Informants were initially asked questions to characterize their socioeconomic profile, such as name, age, gender, profession, marital status, education, and income. We then employed the free list technique [18], in order to extract information on the community's knowledge of medicinal plants. At this point, the informants were asked to list all the medicinal plants they knew.

We also employed the new reading technique [18], which consisted of conducting a slow and detailed reading of the list of plants cited by the informant during the free listing, with the intention of stimulating the informant to remember plants that they had not yet cited. After the listing of all known plants, the interview was continued in order to collect additional data, such as the part of the plant used, forms of use, effectively used plants, preferred plants, and therapeutic indications.

Plant identification was done using literature, comparisons with herbarium specimens, and consultation with experts. A voucher specimen of each recorded species was collected and deposited in a herbarium. All plants mentioned in the interviews were classified as “apparent” (shrubs and trees) and “non-apparent” (herbaceous), according to the woody stem. Moreover, they were also categorized as native or exotic, according to their biogeographical origin; we considered exotic plants species to be those of extracontinental origin.

The local categories of diseases occupied by plants were grouped into 17 categories of body systems, as proposed by the World Health Organization (WHO), based on the work of Almeida and Albuquerque [19]. In this study, we consider local categories of diseases to be the diseases indicated by the informants, according to their own concepts of diseases, which are handled locally through medicinal plants.

### 2.3. Data Analysis

In order to verify the role of “apparent” and “non-apparent” plants, as well as native and exotic species in the local pharmacopoeia, we compared average salience values using the Mann–Whitney test, taking into consideration the most salient plants of the free list as those most important in the local pharmacopoeia [20]. To calculate salience, we used the Anthropac software version 4.0 [21], which took into account the order and frequency of the plants cited in the free list. In addition, we compared the numbers of local categories of diseases occupied by “apparent” and “non-apparent” plants with the chi-square test using Bioestat 5.0 [22].

To address questions regarding the importance of exotic plants in the studied pharmacopoeia, we compared the average salience of native and exotic plants cited in the free list and the number of local categories of diseases occupied by native and exotic plants using the *G* test (contingency table).

## 3. Results

### 3.1. General Characterization of the Pharmacopoeia

Local people cited 71 ethnospecies, sixty-six of which were identified to the genus level and belonged to 35 families (Table 1). The families with the greatest number of named species were Fabaceae (6 species), Lamiaceae and Myrtaceae (5), and Asteraceae (4). The most salient species of the free list were* Eugenia uniflora* L.,* Citrus sinensis *(L.) Osbeck,* Anadenanthera colubrina* (Vell.) Brenan., and* Cereus jamacaru* DC (mandacaru) (Table 1). Ninety-four local categories of diseases were cited, of which the cough and flu were most frequent. Out of the 17 categories of body systems used to group popular therapeutic applications, thirteen were cited by the Engenho Cuieiras informants (Table 1).

### 3.2. Apparent and Non-Apparent Plants

There were significantly more “non-apparent” (herbaceous; 39 sp.; 59%) than “apparent” (shrubs and trees; 27 sp.; 41%) species within the list of 66 species identified to the genus level (*x*^2^ = 2.18; *p* = 0.01).

In relation to the salience, there was a significant difference between “non-apparent” (mean = 0.04; standard error = 0.01) and “apparent” (mean = 0.02; standard error = 0.007) plant species (*Z* (*U*) = 2.0; *p* = 0.042). Accordingly, herbaceous plants were generally cited at the beginning of the free list and have greater citation frequency among informants. The number of local disease categories addressed by the two groups of plants was significantly different (chi-square = 9.564; *p* = 0.003); “non-apparent” species are responsible for treatment of the a greater number of local disease categories (51) than “apparent” species (28). “Non-apparent” species were reported to treat 34 unique local categories of diseases while “apparent” species were exclusive to 10 categories, with significant differences between these two groups of plants (*G* test = 7.203; *p* = 0.014). Some body systems treated only by “non-apparent” species were the control of blood pressure, diabetes, some types of pain, and parasitic diseases, while “apparent” species were used for the treatment of various types of inflammation, cancer, and respiratory problems.

### 3.3. Native and Exotics Plants

The majority of species cited by the informants were exotic (42 species; 63.6%). The species most commonly cited were* Cymbopogon citratus* (DC) Stapf. (27.3%),* Mentha piperita* L. (21.2%), and* Psidium guajava* L. (19.7%). Twenty-four native species were cited (36.4%), of which the most commonly cited were* Eugenia uniflora* L. (19.7%),* Schinus terebinthifolius* Raddi (15.2%), and* Anadenanthera colubrina* (Vell.) Brenan (6.1%). There were significant differences between the numbers of exotic and native plants cited (*x*^2^ = 4.91; *p* = 0.03).

Regarding the relative importance of native and exotic plants in the studied pharmacopoeia, no significant differences were found between average salience values of native (mean = 0.02; standard error = 0.009) and exotic (mean = 0.03; standard = 0.01) plants (*Z* (*U*) = 0.632; *p* = 0.263). That is, the order and frequency of citations did not differ between native and exotic plants.

Exotic species exhibited a significantly greater richness of local categories of diseases (61) than native (30) species (*G* test = 4.8; *p* = 0.028). A total of 43 diseases were treated only by exotic species, while native plants exclusively treated 12 diseases. These values showed a significant difference (*G* test = 8.5; *p* = 0.003). Body systems only treated by exotic species were infectious parasitic diseases, neoplasms, and disorders of the nervous system, while native plants do not exclusively treat any body system.

## 4. Discussion

### 4.1. Apparent and Non-Apparent Plants

The predominance of herbaceous species in the studied pharmacopoeia may be related to the fact that most of them were obtained from anthropogenically disturbed areas, secondary vegetation, or homegardens [23–25]. These herbaceous plants grow rapidly, disperse easily, and are small [26]; thus, they require only a small area for growth and are better adapted to disturbed environments, such as the fragmented Atlantic Forest landscape dominated by a sugarcane monoculture found in the present study.

In a survey of medicinal plants, Stepp and Moerman [23] also found a higher incidence of herbaceous species. These authors explain this finding due to the fact that medicinal plants need to be abundant and affordable. Therefore, species that are close to communities or species that were taken from more distant sites and transplanted to grow near homes are preferred, and this explains why herbs have greater representation in different medicinal floras, as was also found by Heinrich and Barrera [27] in a community in Oaxaca Mexico.

One may also speculate that the predominance of herbaceous species in local pharmacopoeia is due to the greater amount of bioactive compounds found in this group of plants, which would follow one of the predictions of the ecological apparency hypothesis from a chemical view point [6]. Similar results were found by Lozano et al. [28], with herbaceous species also possessing the greatest richness of ethnospecies in a pharmacopoeia from a community located close to Cerrado vegetation. These authors also attributed this observation to the ecological apparency hypothesis. For example, comparing “apparent” and “non-apparent” plants in a literature review, we found that “non-apparent” plants are frequently observed in local pharmacopoeia; generally these plants are more cultural and have more bioactive compounds and a higher occurrence of secondary metabolites of low molecular weight [29]. Thus, the inclusion hypothesis explains that the occurrence of plants in a local pharmacopoeia may be also associated with environmental factors, such as chemicals, thus demonstrating that this phenomenon cannot be explained by just one factor.

Moreover, in the present study most of the herbaceous species that treat unique diseases are exotic plants (32 sp.; 82%), as was also observed by dos Santos et al. [16]; therefore, the diversification hypothesis becomes relevant (see [1]). The survey data suggests that the introduction of exotic species into the local pharmacopoeia of a studied community may involve the need for therapeutic diversification, or the desire to extend the spectrum of treated disorders in the community by using exotic plants [3, 30]. Thus, the phenomenon of acculturation, or the loss of knowledge, discussed by Caniago and Siebert [12] can be seen as an adaptive response to environmental changes and/or a search for new chemical compounds [13] and not simply a process of loss of knowledge.

Exotic plants have a strong influence on the pharmacopoeia of the present study because these plants are predominantly herbaceous and are important in disturbed areas. Furthermore, exotic plants are as versatile as native plants in the treatment of diseases [28], as will be discussed further in the next section.

### 4.2. Native and Exotic Plants

The richness of exotic species cited by informants in the present study, and observed, for example, in the work of Alencar et al. [3], can be explained by the factors mentioned above, such as processes of landscape transformation that favored their establishment, and consequently greater availability of these species in the studied environment. For example, the native species cited by informants are currently being underused by the community because, according to ancient residents, many of these plants were once easily accessible, but are currently difficult to find in the region. Due to expanding agriculture and increased urban construction in the region, many areas of native vegetation have been eliminated, forcing the local population to seek alternative resources to treat their diseases. These alternatives include the incorporation of exotic and herbaceous species into their medicinal repertoire, thus reinforcing the argument that landscape changes affect the composition of the local pharmacopoeia. Furthermore, Giovannini et al. [31] suggest, for example, that the complementary use of plants for the treatment of diseases along with allopathic medicine is a result of global environmental changes.

It should be noted that some biased conclusions have been published that seek to explain the presence of exotic species in traditional medical systems as a passive process of acculturation, which is promoted by the growth and development of urban centers and the stagnation of rural areas [9, 12]. However, the increased use of exotic species may indeed promote a wider variety of uses that, in some cases, treat diseases that native species do not (see [1, 3]), thus, providing an evolutionary advantage to human groups that include such plants into their pharmacopoeia [13]. For example, in this study we found that in fact exotic plants treat a wider range of local categories of diseases than native species, which was also observed by Janni and Bastien [32] in studies on the pharmacopoeia of Kallawaya in India, where exotic plants were used to treat diseases of a greater number of body systems.

Another aspect of the role of exotic species in local pharmacopoeia is that they are often used to treat specific diseases that native species do not and, therefore, fill therapeutic gaps left by native species [33]. In addition, studies have suggested that exotic species have the advantage of increased palatability [1, 2], which would increase their versatility and benefit of their consumption, both medically and nutritionally.

## 5. Final Considerations

In conclusion, the studied pharmacopoeia has been influenced by landscape changes, since there was a predominance of herbaceous exotic medicinal species found within the Atlantic Forest. Although linked to changes in vegetation, other factors may be involved in the preference of these species, such as the fact that herbaceous plants contain classes of secondary bioactive compounds, which are only present in small quantities in woody plants, as postulated by the hypothesis of ecological apparency. Regarding exotic species, their inclusion should not be simply considered acculturation, since the exotic plants of the studied community also appear to be incorporated into the medical repertoire in an attempt to meet demands for specific local categories of diseases that native plants do not fill, which fulfills some of the assumptions of the diversification hypothesis. Thus, we recognize a possible adaptation of the studied rural community, which could be a survival advantage in the context of environmental change. Furthermore, the patterns of human behavior and knowledge of biological resources suffer different pressures, demonstrating that the phenomenon of incorporating medicinal plants into local pharmacopoeia is complex and consists of a combination of several factors.

## Figures and Tables

**Table 1:** Medicinal plants cited by residents of the Engenho Cuieiras community in the city of Aliança, Pernambuco, Northeastern Brazil. ADND = not defined disorders or pain; DGENM = endocrine, nutrition, and metabolic diseases; DIP = infectious and parasitic diseases; DPTCSC = diseases of the skin and subcutaneous tissue cell; DSOTC = disease of the musculoskeletal system and connective tissue; IG = general inflammations; NEO = neoplasms; TSC = circulatory system disorders; TSSO = sensory system disorders (eye/ear); TSD = digestive system disorders; TSGU = genito-urinary system disorders; TSN = disorders of the nervous system; TSR = disorders of the respiratory system.

Family/scientific name	Popular name	Origin	Habit	Therapeutic indication	Body system
*Acanthaceae*					
*Justicia gendarussa* Burm.F.	Anador	Exotic	Herb	Pain	ADND, TSD
*Adoxaceae*					
*Sambucus australis* Cham. & Schltdl.	Flor de Sabugo	Native	Three	Cough	TSR
*Amaranthaceae*					
*Pfaffia glomerata* (Spreng.) Pedersen	Acônico	Exotic	Herb	Fever	ADND
*Anacardiaceae*					
*Schinus terebinthifolius* Raddi	Aroeira	Native	Three	Injury, general inflammation, inflammation in uterus	IG, DTCSC, TSGU
*Anacardium occidentale* L.	Cajú-Roxo	Native	Three	Injury, general inflammation, intestinal infection	DTCSC, IG, TSD
*Apiaceae*					
*Foeniculum vulgare* Mill.	Êndio	Exotic	Herb	Pain, intestinal problem	ADND, TSD
*Coriandrum sativum L.*	Coentro	Exotic	Herb	Stomaching	TSD
*Pimpinella anisum* L.	Erva-doce	Exotic	Herb	Soothing, intestinal problem	TSN, TSD
*Arecaceae*					
*Syagrus *sp.	Coco-catolé	Native	Three	Osteoporosis	DSOTC
*Asteraceae*					
*Gymnanthemum amygdalinum* (Delile) Sch.Bip. ex Walp.	Alcachofra	Exotic	Herb	Pain of belly, liver problems	TSD, TSD
*Matricaria recutita *L.	Camomila	Exotic	Herb	Soothing	TSN
*Acanthospermum hispidum *DC.	Espinho-de-cigano	Exotic	Herb	Catarrh, cough	TSR
*Conyza bonariensis *(L.) Cronq.	Rabo-de-raposa	Exotic	Herb	Fungus	DPTCSC
*Bignoniaceae*					
*Handroanthus impetiginosus* (Mart. ex DC.) Mattos	Pau-d'arco-roxo	Native	Three	Inflammations	IG
*Burseraceae*					
*Protium heptaphyllum *(Aubl.) Marchand	Amescla, amescla- roxa e amescla-branca.	Native	Three	Pain, internal inflammations, intestinal infection	ADND, IG, TSD
*Cactaceae*					
*Cereus jamacaru* DC	Mandacaru, Cardeiro	Native	Shrub	Reins, spinal problems	TSGU, DSOTC
*Chenopodiaceae*					
*Chenopodium ambrosioides *L.	Mastruz	Exotic	Herb	Catarrh, bronchitis, worms	TSR, DIP
*Costaceae*					
*Costus spicatus *Sw.	Cana-de-macaco	Exotic	Herb	Blood pressure, spinal problems, reins	TSC, DSOTC, TSGU
*Cucurbitaceae*					
*Sechium edule *(Jacq.) Sw.	Chuchu	Exotic	Herb	Blood pressure	TSC
*Cucumis sativus *L.	Pepino	Exotic	Herb	Blood pressure	TSC
*Euphorbiaceae*					
*Phyllanthus niruri *L.	Quebra-pedra	Exotic	Herb	Reins, renal problem	TSGU, TSGU
*Cnidoscolus urens *(L.) Arthur	Urtiga-branca	Native	Herb	Infections, eyes	IG, TSSO
*Jatropha gossypiifolia *L.	Pinhão-roxo	Native	Herb	Heals scarring	DPTCSC
*Fabaceae*					
*Stryphnodendron adstringens *(Mart.) Coville	Barbatimão	Native	Three	Wounds/cuts	DPTCSC
*Hymenaea courbaril *L.	Jatobá	Native	Three	Catarrh, cough	TSR
*Mimosa pudica *L.	Malícia-fina	Native	Herb	General inflammation, inflammation of the uterus, swelling of the legs	IG, TSGU, TSC
*Senna occidentalis *(L.) Roxb.	Manjerioba	Exotic	Herb	Catarrh, cough	TSR
*Bowdichia virgilioides *Kunth	Sucupira	Native	Three	Internal inflammations	IG
*Bowdichia virgilioides *Kunth	Sucupira-branca	Native	Three	Pain	ADND
*Lamiaceae*					
*Rosmarinus officinalis *L.	Alecrim	Exotic	Herb	Fever	ADND
*Plectranthus amboinicus *(Lour.) Spreng	Hortelã-Graúda	Exotic	Herb	Catarrh, cough	TSR
*Ocimum basilicum* L.	Manjericão	Exotic	Herb	Antiabortive, infections	IG, TSGU
*Mentha piperita *L.	Hortelã-miúda	Exotic	Herb	Anti-VCA, blood pressure, flu, parasites, amebiasis, pain in the belly	TSC, TSR, DIP, TSD
*Ocimum campechianum *Mill.	Alfavaca-do-mato	Native	Herb	Control of rates, gases, prostate	DGENM, TSD, TSGU
*Lauraceae*					
*Persea americana *Mill.	Abacate	Exotic	Three	Renal problem	TSGU
*Nectandra cuspidata *Ness & Mart.	Canela	Native	Three	Numbness, inflammation	TSD, IG
*Liliaceae*					
*Aloe vera *(L.) Berm.f.	Babosa	Exotic	Herb	Skin problems, inflammation, gastritis, osteoporosis	DPTCSC, IG, TSD, DSOTC
*Allium cepa *L.	Cebola-branca	Exotic	Herb	Catarrh	TSR
*Malpighiaceae*					
*Malpighia glabra *L.	Acerola	Exotic	Shrub	Cough	TSR
*Malvaceae*					
*Abelmoschus esculentus *L.	Quiabo	Exotic	Herb	Diabetes	DGENM
*Urena lobata *L.	Malva-Rosa	Native	Herb	Pain, intestinal problem	ADND, TSD
*Mimosaceae*					
*Anadenanthera colubrina* (Vell.) Brenan.	Angico	Native	Three	Catarrh, cough	TSR
*Monimiaceae*					
*Peumus boldus *Mol.	Boldo	Exotic	Herb	Intestinal problems	TSD
*Moraceae*					
*Ficus *sp.	Figo	Exotic	Three	Fungus	DPTCSC
*Myrtaceae*					
*Eugenia uniflora *L.	Pitanga	Native	Shrub	Intestinal problems	TSD, TSD
*Psidium guajava *L.	Goiaba	Native	Three	Intestinal problems	TSD, TSD
*Eucalyptus citriodora *Hook.	Eucalipto	Exotic	Three	Respiratory problems	TSR
*Psidium guineense *S.W.	Araçá	Native	Three	Intestinal problems	TSD
*Syzygium jambolanum *(Lam.) DC.	Azeitona	Exotic	Three	Diabetes	DGENM
*Oxalidaceae*					
*Averrhoa carambola *L.	Carambola	Exotic	Three	Renal problems	TSGU
*Pedaliaceae*					
*Sesamum orientale* L.	Gergilin	Exotic	Herb	Pain	ADND
*Poaceae*					
*Cymbopogon citratus *(DC) Stapf.	Capim-santo	Exotic	Herb	Pain, intestinal problems, soothing, pain	ADND, TSD, TSN, DSOTC
*Saccharum officinarum *L.	Cana-de-açucar	Exotic	Herb	Blood stagnation	TSC
*Punicaceae*					
*Punica granatum *L.	Romã	Exotic	Shrub	Inflammation of the throat, intestinal infections	IG, TSD
*Rosaceae*					
*Malus domestica *Borkh.	Maçã	Exotic	Shrub	Vision	TSSO
*Rubiaceae*					
*Morinda citrifolia *L.	Noni	Exotic	Shrub	Cholesterol, diabetes, pain, rheumatic fever, cancer prevention	DGENM, ADND, DIP, NEO
*Genipa americana *L.	Jenipapo	Native	Three	Catarrh	TSR
*Borreria verticillata *(L.) G. Mey.	Vassoura-de-botão	Exotic	Herb	Pain, inflammations, cough	ADND, IG, TSR
*Rutaceae*					
*Ruta graveolens *L.	Arruda	Exotic	Herb	Headache, earache	ADND, ADND
*Citrus sinensis *(L.) Osbeck	Laranja	Exotic	Three	Soothing, insomnia	TSN
*Solanaceae*					
*Solanum paniculatum *L.	Berinjela	Exotic	Herb	Cholesterol	DGENM
*Solanum paniculatum *L.	Jurubeba-branca, Jurubeba-roxa	Exotic	Herb	Catarrh, inflammations, stomachache, blood	TSR, IG, TSD, DGENM
*Physalis angulata *L.	Camapu	Native	Herb	Skin problems	DPTCSC
*Verbenaceae*					
*Lippia alba *(Mill.) N. E. Br.	Herb-cidreira	Exotic	Herb	Digestive problems, pain, insomnia, soothing	TSD, ADND, TSN, TSN
*Vitaceae*					
*Cissus verticillata *(L) Nicolson & C.E. Jarvis	Insulina	Native	Herb	Diabetes	DGENM
*Zingiberaceae*					
*Alpinia speciosa *(Blume) D. Dietr.	Colônia	Exotic	Herb	Fever, sinusitis, catarrh, flu, headache	ADND, TSR, TSR, ADND
